# Clustered Gene Expression Changes Flank Targeted Gene Loci in Knockout Mice

**DOI:** 10.1371/journal.pone.0001303

**Published:** 2007-12-12

**Authors:** Luis M. Valor, Seth G. N. Grant

**Affiliations:** Genes to Cognition Programme, The Wellcome Trust Sanger Institute, Cambridge, United Kingdom; Tufts University, United States of America

## Abstract

**Background:**

Gene expression profiling using microarrays is a powerful technology widely used to study regulatory networks. Profiling of mRNA levels in mutant organisms has the potential to identify genes regulated by the mutated protein.

**Methodology/Principle Findings:**

Using tissues from multiple lines of knockout mice we have examined genome-wide changes in gene expression. We report that a significant proportion of changed genes were found near the targeted gene.

**Conclusions/Significance:**

The apparent clustering of these genes was explained by the presence of flanking DNA from the parental ES cell. We provide recommendations for the analysis and reporting of microarray data from knockout mice

## Introduction

Microarray technology has been widely assimilated by gene expression researchers and its success is largely due to its power of assaying thousands of transcripts in a genome wide manner. One of the most popular experimental designs has been to determine the gene expression changes that occur in knockout mice where it is correlated with phenotypic changes. The usual objective is to obtain insights into the role of the mutated protein by identifying particular genes under its control, and retrieve candidate genes that may explain the altered phenotype in the mutant animal.

The creation of knockout mice involves the modification of the genome of mouse embryonic stem (ES) cells through a variety of processes including homologous recombination or other mutagenic procedures [1]. The majority of gene targeting experiments have been performed in ES cells derived from the 129 strain of mice. Following germ-line transmission of the mutant allele, the founder mice are often backcrossed onto other strains, creating hybrid animals. The relative contribution of the genetic background of the ES cell and the strain to which it was bred has been widely studied [2].

We have studied the effects of knockout (KO) mutations on the mechanisms of learning and memory [3], [4]. Patterns of neural activity induced by learning or artificial stimuli lead to activation of signal transduction pathways, which ultimately lead to the cellular changes underlying learning and memory. The two major cellular changes involve strength of synaptic transmission (synaptic plasticity) and gene expression (activity-dependent gene expression). It is clear from studies in the sensory-motor synapses of *Aplysia californica* and the hippocampus of mice and rats that neurotransmitter receptors initiate the signalling events that lead to both synaptic plasticity and activity-dependent gene expression [5], [6], [7], [8], [9], [10], [11]. L-glutamate is the dominant excitatory neurotransmitter in the central nervous system of vertebrates and glutamate receptors are of special relevance to synaptic plasticity. The ionotropic *N*-methyl-D-aspartate (NMDA) and metabotropic (mGLuR) subtypes of glutamate receptor are commonly involved in experimental plasticity-driven transcriptional changes. Both the NMDA and mGluR receptors are connected by a series of adaptor proteins [12], [13] and can be physically isolated from mouse brain in ∼2000-kDa multiprotein complexes called NRC or MASC (NRC, NMDA Receptor Complex; MASC, MAGUK Associated Signaling Complex) [14], [15], [16], [17]. NRCs are organized into receptor, adaptor, signalling, cytoskeletal and novel proteins, and includes cell adhesion molecules, kinases, phosphatases, GTPase-activating proteins and Ras and MAPK pathway components. NRC/MASC form a subset of the post-synaptic density which is thought to be comprised of a set of complexes. Such a complex offers an attractive model for the organisation and coordination signalling networks [14], [17], [18]. It was hypothesised that the function of the NRC is to orchestrate the signalling pathways downstream of receptor activation and coordinate both synaptic plasticity and activity-dependent gene expression amongst other changes.

We were specifically interested in the previously characterized KO mice for PSD-95 and SAP102 [3], [4]. Both proteins are from the MAGUK (Membrane Associated GUanylate Kinase) family, which members share a common structure of three PDZ (PSD-95/Dlg/ZO-1), an SH3 (Src-homology-3) and guanylate kinase (GK) domains and together enable protein-protein interactions. The postsynaptic density 95 (PSD-95) protein is a homolog of the *Drosophila* suppressor gene, *dlg* [19] and acts as a scaffold to organize postsynaptic proteins into signalling complexes [4] or anchoring complexes for glutamate receptors and cytoskeletal proteins, as reviewed elsewhere [12]. Mice lacking PSD-95 or SAP102 were found to have enhanced forms of NMDA receptor-dependent long-term potentiation [3], [4], [20], [21] and exhibited impairments in spatial learning (and other forms of behavioural plasticity) [22], [23], [24]. Our original aim was to identify those transcripts that were controlled by PSD-95 and SAP102 and therefore try to track their dependent signalling pathways. Surprisingly, and because of the unbiased nature of microarray analyses, we found that the most significantly changing genes were clustered in the neighbourhood of the KO gene. We observed that this phenomenon was frequent in KO studies and, therefore, special care should be taken in the interpretation of this kind of experiments.

## Results

To determine the gene expression changes in the hippocampus of mice lacking SAP102 or PSD-95, total RNA was isolated from the hippocampus of SAP102^−/Y^ and PSD-95^−/−^ mutant mice and littermate wild-types. RNA was hybridized to MG-430 2.0 arrays (Affymetrix) that contained 45,037 probe sets excluding controls. The data was normalized and filtered (see Materials and Methods). We first examined the expression data from SAP102 mutants and found that 65% (13/20) of the significantly changed probe sets were encoded by chromosome X, which also encodes SAP102. This unexpected finding suggests that either SAP102 preferentially regulates genes on the X-chromosome or that there is some *cis*-acting effect of the mutation.

**Figure 1:**
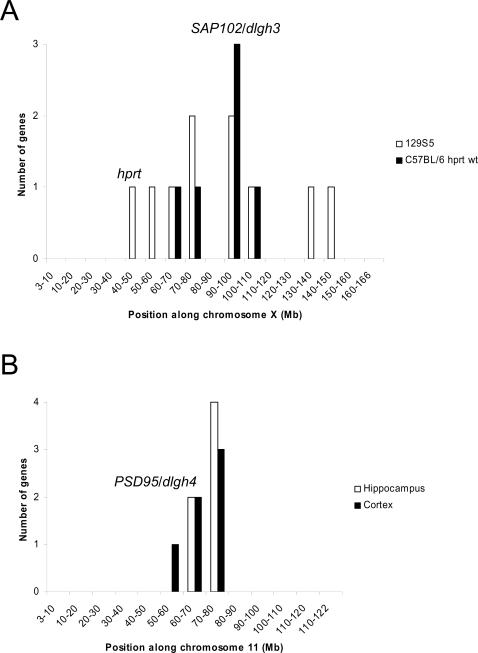
Clustering of changed genes around *SAP102/dlgh3* and *PSD-95/dlgh4* loci. *A*, significant genes (x-axis) from SAP102^−/Y^ hippocampus along chromosome X (y-axis), indicating the location of SAP102 gene (*dlgh3*) and *hprt.* White histogram, results from 129S5 strain animals; black histogram, results from C57BL/6j after selection of *hprt* deletion (see text). *B*, probe sets from PSD-95^−/−^ along chromosome 11, where the position of the mutated gene (*dlgh4*) is indicated. White histogram, hippocampus mRNA; black histogram; cortex mRNA.

We focused on the SAP102 regulated genes on the X-chromosome and found they were clustered around the SAP102/*dlgh3* gene (Figure 1). These genes spanned a region of ∼98 Mb (Figure 1A, Table 1). Within this cluster was the *hprt* (hypoxanthine guanine phosphoribosyl transferase) gene, a selection marker extensively used in ES cells to generate transgenic mice [25], [26], [27], [28]. In our case, the ES cell clone used to generate our knockout mice was E14TG2a HM-1, which harbours an *hprt* allele containing a 55 kb-deletion in its 5′-flanking region [29]. Using a PCR protocol specifically designed to genotype this deletion [30], we found it was correlated with the reduction of HPRT expression (data not shown). The *hprt* and SAP102/*dlgh3* loci are separated by ∼48 Mb and since the *hprt* locus of 129S5 mice does not contain the deletion, these data suggested that the observed changes in *hprt* gene expression in SAP102 mutant mice was the result of a linked mutation originated in the ES cells. Following gene targeting of SAP102/*dlgh3* gene in E14TG2a ES cells (129P2 background), the chimera was crossed to 129S5 and subsequently backcrossed. This provided an important clue that genetic background of the ES cells might be contributing to the gene expression profiling.

**Table 1:** List of significant gene expression probe sets from X chromosome in SAP102^−/Y^ samples.

Affy ID	Gene symbol	Access No	Gene description	Fold change	Chromosomal location
1448736_a_at	Hprt	NM_013556	hypoxanthine guanine phosphoribosyl transferase	0.0341	49265731
1438737_at	Zic3	AV029604	zinc finger protein of the cerebellum 3	0.467	54382836
1456746_a_at	Cd99l2	BB334959	Cd99 antigen-like 2	0.273	67681618
*1420357_s_at*	Xlr3a	NM_011726	X-linked lymphocyte-regulated 3a	3.586	69339194 and other loci
**1442882_at**	3′ to Fundc2	BB315069	leucine zipper, putative tumor suppressor 2	0.406/0.412	71651825
1453246_at	Rab39b	AK020665	RAB39B, member RAS oncogene family	2.062	71826931
*1427985_at*	9630042H07Rik	BC027796	RIKEN cDNA 9630042H07 gene	1.881	91225268
**1416918_at**	Dlgh3	NM_016747	discs, large homolog 3 (Drosophila)	0.113/0.113	97020543
**1439300_at**	Chic1	BG065782	cysteine-rich hydrophobic domain 1	0.402/0.307	99593901
**1418774_a_at**	Atp7a	U03434	ATPase, Cu++ transporting, alpha polypeptide	0.474/0.472	102327361
1418141_at	Dcx	BB418548	doublecortin	0.515	139103842
1438575_a_at	genomic	BG143413	Adult male hippocampus cDNA, RIKEN full-length enriched library, clone:2900056M20 product	0.357	147637637
1438576_x_at	genomic	BG143413	Adult male hippocampus cDNA, RIKEN full-length enriched library, clone:2900056M20 product	0.476	147637638

Affy IDs in bold are probe sets that appeared in 129S5 and C57BL/6 *hprt* wild-type; Affy IDs in italic are probe sets only appearing in C57BL/6 *hprt* wild-type. The difference in probe sets were likely due to differences in the statistical power of both analyses (ten 129S5 samples *vs.* seven C57BL/6 samples). When two numbers appear in the “Fold change” column, the upper number is the fold change obtained from 129S5 samples and the lower number from C57BL/6 samples. Chromosomal location denotes the first nucleotide position of the probe set (Ensembl release 45).

To further explore this hypothesis, we examined the degree of linkage between both loci. Using the MGI database (www.informatics.jax.org) we estimated the percentage of independent segregation of hprt and *SAP102/dlg3* to be 27% (27 cM). We tested this using a cross between *SAP102/dlg3* heterozygous females and 129S5 (wild type) males, which produced 37 litters, and subsequent genotyping. Only 27% of the heterozygous females had two wild-type alleles of *hprt* and 29% of E17.5 male embryos showed segregation of *hprt* and *SAP102/dlg3* mutations. These data are in keeping with the conclusion that the entire portion between both loci originated in the original E14TG2a ES cells.

**Figure 2:**
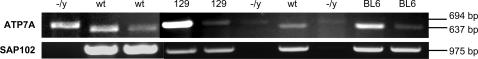
PCR genotyping results for Atp7a and Dlgh3 genomic regions. Two bands were observed for Atp7a reactions, one of 694 bp corresponding to 129-derived sequences and another of 637 bp corresponding to C57Bl6-derived sequences (upper panel). The appearance of the 694 bp-band coincided with the presence of the SAP102 mutation (-/y) (lower panel). Wild-type 129S5 (129) and C57BL/6j (BL6) were used as positive controls.

These observations suggest that further backcrossing and marker selection would result in a reduction of the E14TG2a DNA. To explore this possibility we analyzed microarray data from SAP102^−/Y^ hippocampi that contained the wild-type allele of *hprt* mutation. In this experiment we used SAP102^−/Y^ mutant mice that had been backcrossed 3 generations onto the C57BL/6j background. In this case, only six probe sets were changed from the X chromosome (∼29% of the significant probe sets; 6/21) and spanned a shorter region of ∼48 Mb (Figure 1A, Table 1). This clustering was significantly different from a random distribution along the X chromosome (*P*<0.05, χ^2^ = 26.6 d.f. = 16). Four of the five probe sets were common to the previous experiment (Table 1) and the change was similar in direction and magnitude, thus reproducing the effect of the ES cell-derived sequences and suggesting changes were independent of the host backcrossing genetic background. Since we used C57BL/6j animals for the new analysis, we could take advantage of the availability of primers that distinguished C57- and 129-derived strains genotypes. We focused on *Atp7a*, which expression was changed in the microarray experiment and is found ∼5 Mb from *SAP102/dlg3* and tested if it was of 129 or C57BL/6 origin. Using a published genotyping protocol (in which the amplicon covers exon 8 of Atp7a [31]), we found that the all samples that showed changes in Atp7a expression were of 129 origin (Figure 2). These data further support the conclusion that the changes in gene expression in the mutant mice involving the chromosome X can be explained by genetic background effects.

Similar effects were observed for other mutations involving autosomes. We took advantage of the results obtained in another knockout for a MAGUK protein, PSD-95, which is encoded on chromosome 11. Using an identical microarray platform to that used for SAP102 mutants, we found that ∼47% (8/17) of the significantly changed probes in hippocampus were encoded on chromosome 11. As shown in Figure 1B there was significant clustering of probe sets around the *dlgh4* locus and spanned a region of ∼5 Mb (*P*<0.001, χ^2^ = 33.7 d.f. = 11). This shorter length of the ES cell-derived region in PSD-95 mutants may be due to the larger number of backcrosses (>10 in PSD-95 onto a C57BL/6 background) compared to the SAP102 mutants (∼48 Mb after 3 backcrossed on C57BL/6). These data are consistent with the view that the number of backcrosses helps to shorten the length of ES cell-derived sequences as well as marker selection (*hprt* in the SAP102 mutants) and should be considered in microarray profiling experiments in knock-out animals. The above data is based on studies of hippocampus transcript levels and we asked if similar results would be observed in other regions of the nervous system. Using cortex RNA from PSD-95 mutants we found that 4 of the 6 probe sets that were significant in the hippocampus were also significant in the cortex of the same animals (Table 2), thus reproducing the effects of ES-cell derived sequences in gene expression. For further details on the statistical analysis of both SAP102 and PSD-95 mutants, see Tables 3 and 4.

**Table 2:** List of significant gene expression probe sets on chromosome 11 from PSD-95^−/−^ mutants.

Affy ID	Gene symbol	Access No	Gene description	Fold change	Chromosomal location
*1459880_at*	Agxt2l2 intron	BB711284	RIKEN cDNA 2900006B13 gene	1.808	51439643
*1442019_at*	Glp2r intron	BB627097	Recoverin	1.90	67503392
**1424893_at**	Ndel1	BC021434	nuclear distribution gene E-like homolog 1 (A. nidulans)	0.0362/0.216	68645519
**1419581_at**	Dlgh4	AI646416	discs, large homolog 4 (Drosophila)	0.223/0.209	69858787
**1419580_at**	Dlgh4	AI646416	discs, large homolog 4 (Drosophila)	0.262/0.186	69861220
1459105_at	3′ to Dlgh4	BB102018	Transcribed sequences	0.541	69863152
1443698_at	Fbxo39	BB645745	F-box protein 39	0.096	72119566
**1443621_at**	3′ to Fbxo39	BG092359	Transcribed sequences	15.87/18.72	72129544
**1436943_at**	Cyb5d2	BB078068	cytochrome b5 domain containing 2	0.25/0.385	72593471
*1441404_at*	Pafah1b1 intron	BG075643	H3149G01-3 NIA Mouse 15K cDNA clone H3149G01 3′.	0.115	74522814
**1456947_at**	Pafah1b1 intron	BG072758	Transcribed sequences	0.333/0.361	74526997

Affy IDs in bold are probe sets that appeared in hippocampus and cortex; Affy IDs in italic are probe sets only appearing in cortex. The difference in probe sets within both tissues was likely due to differences in the statistical power of both analysis (9 hippocampal samples *vs.* 4 cortex samples). When two numbers appear in the “Fold change” column, the upper number is the fold change obtained from hippocampus and the lower number from cortex. Chromosomal location denotes the first nucleotide position of the probe set (Ensembl release 45).

**Table 3:** Distribution of significant genes within the chromosome of the ablated gene.

	χ^2^ values	Region (Mb)	Region (cM)	*P*-values	% genes
**Dlgh3-129S5**	12.8	98	50	3·10^−3^	100% (10/10)
**Dlgh3-C57BL**	26.6^*^	48	15	6·10^−4^	100% (6/6)
**Dlgh4**	33.7^****^	5	5	3·10^−6^	100% (6/6)
**Foxa3**	31.1^*^	28	20	10^−4^	100% (8/8)
**Ercc1^†^**	12.8	60	30	3·10^−2^	72.7% (8/11)
**Neb^†^**	9.9	75	40	7·10^−3^	53.3% (8/15)
**Efemp2^†^**	7.5	12	10	5·10^−3^	80.0% (4/5)

χ^2^ tests were performed in the available mutants under the null hypothesis of random distribution (see Materials and Methods): **, p<0.05; ***, p<0.01; ****, p<0.001. *P*-values were calculated comparing the region containing the significant genes with the rest of the chromosome (Student's t-tests). ^†^, when the clustering did not allow the test or the test failed, the most significant region was calculated using different sizes of genomic region by trial-and-error. Region of significance is expressed in Mb and the correspondent cM. The % of genes contained therein is also expressed.

**Table 4:** Distribution of significant genes between all the chromosomes.

	Random %	Norm. 1%	Norm. 2%	Other chrom.	Total genes
**Dlgh3-129S5**	85.35^****^	90.84^****^	88.11^****^	No	19 (52.63%)
**Dlgh3-C57BL**	66.05^**^	75.14^****^	73.10^****^	No	21 (33.33%)
**Dlgh4**	74.73^****^	55.03^****^	75.16^****^	No	12 (50.00%)
**Foxa3**	91.25^****^	85.32^****^	92.21^****^	No	10 (80.00%)
**Ercc1**	50.22	8.93	40.64	No	92 (11.96%)
**Neb**	10.05^*^	5.77	21.19^****^	Chr. 9	140 (10.71%)
**Efemp2**	57.42^****^	43.33^*^	22.59^*^	No	34 (14.71%)

χ^2^ tests were performed in the available mutants under the null hypothesis of random distribution: Random, equal proportion; Norm.1, proportion normalized by chromosome length; Norm.2, normalized by number of genes in each chromosome (see Materials and Methods): *, p<0.1; **, p<0.05; ***, p<0.01; ****, p<0.001. Percentages are the contribution of the chromosome for each ablated gene to the deviation from each random distribution and were calculated as proportion of the χ^2^ values. If a chromosome other than that encoding the knock-out gene had more contribution is stated in “Other chrom”. Total genes are the number of the retrieved significant genes.

**Figure 3:**
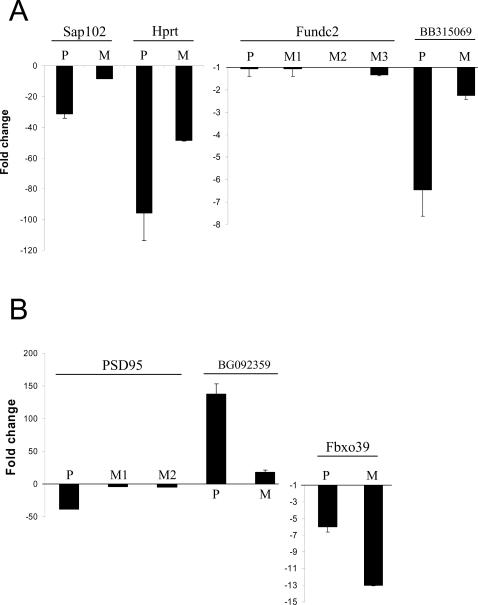
Validation of microarray analyses by qRT-PCR assays in PSD-95 and SAP102 mutants. Hippocampus mRNA was extracted and level of mRNA detected using microarrays or qRT-PCR. *A*, SAP102 mutant mice; results for SAP102, Hprt, Fundc2, BB315069 (3′ to Fundc2) are shown. B. PSD-95 mutant mice; results for PSD-95, Fbxo39 and BG092359 (3′ to Fbxo39). y-axis: fold change of mutant mRNA levels compared to control; mean±s.e.m. P, qRT-PCR data; M, microarray data. M1, M2… refers to different probe sets for the same gene. Note different scale of y-axes.

We also validated the microarray experiments performed in SAP102 and PSD-95 mutants by qRT-PCR. As shown in Figure 3 the changes in gene expression detected with microarrays were confirmed using qRT-PCR for SAP102, HPRT, Fundc2, and BB315069 (3′ to Fundc2) in the SAP102 mutants and PSD-95, Fbxo39 and BG092359 (3′ to Fbxo39) in the PSD-95 mutants.

**Figure 4:**
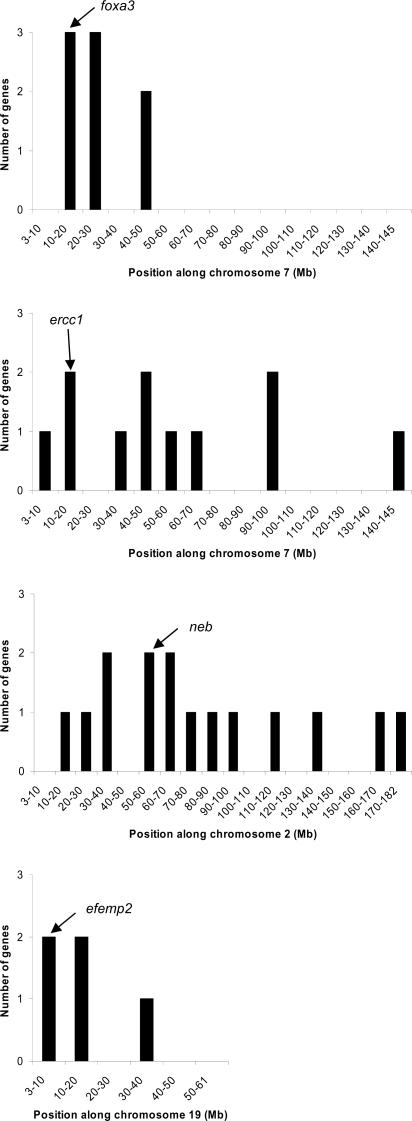
Number of significantly changed genes on targeted chromosome for Foxa3, Neb, Ercc1 and Fibulin-4 mutants. y-axis: number of changed genes, x-axis: chromosomal position. The arrow indicates the location of the mutated genes in each case.

From the above experiments on brain mRNA from SAP102 and PSD-95 mutants it seems likely that these effects of genes linked to the mutant allele might be generally important in other knockout lines and other tissues. To address this issue we analyzed recent data deposited in ArrayExpress (www.ebi.ac.uk/arrayexpress/) that employed the same format of arrays as used in our study. The purpose was not to exhaustively compile the existent data but to provide further examples. Data from four mutant genes were analyzed: Foxa3 [32], Nebulin (*neb*) [33], Ercc1 [34] and Fibulin-4 (*efemp2*) [35] (Figure 4), using RNA derived from testes, skeletal muscle, liver and aorta respectively.

In all four knockouts we found changes in gene expression in genes near to the targeted locus; however the potential boundary between ES cell and host DNA was not clear in all mutants (Table 3, Figure 4). The number of changed genes was plotted for the length of the relevant chromosome and the position of the targeted gene indicated. This allows us to examine clustering ‘within chromosomes’ and to compare with random distributions (see Materials and Methods). For chromosome 7 and the *foxa3* locus, of the 8 genes that changed on this chromosome (including the mutated gene), all were clustered within 28 Mb of the mutation (*P*<0.01, χ^2^ = 31.1 d.f. = 14), despite 11 backcrosses onto C57BL/6 strain [32]. For *ercc1* and *neb* mutants, the changed genes were distributed more widely on chromosome 7 and 2 respectively. Although there was a trend toward clustering with the mutant locus, this distribution was not statistically significant (Table 3): for *ercc1* there were 72.7% of significant genes within ∼60 Mb (∼42% of the chromosome); for *neb* there were 53.3% within ∼75 Mb (∼42% of the chromosome). The inability to identify more conclusive evidence of linkage in *ercc1* or *neb* mutants was probably due to the large number of changed genes: compared to SAP102, PSD-95 and Foxa3 where 21, 12 and 10 genes respectively were changed, 140 genes were changed in *ercc1* mutants and 92 in *neb* mutants (Table 4). In the case of the *efemp2* locus on chromosome 19, there were 4 genes changed; however clustering was not statistically significant (Table 3) perhaps because chromosome 19 is too short and only a small portion (∼8% of the chromosome, which was absent of significant genes) was estimated to segregate independently and thus it is possible that the entire chromosome was derived from ES-cell background. Thus using the ‘within chromosome’ clustering does not reveal statistically significant results for all knockout mice and appears to be influenced by chromosome size and number of significantly changed genes. To overcome these limitations we used an alternative approach where we compared the frequency of changed genes and their contribution to deviation from random distributions on the targeted chromosome with other chromosomes (see Materials and Methods). Using this ‘between chromosome’ method we found that of 7 mutant examined, 6 showed evidence of clustering of changed genes to the targeted chromosome (Table 4).

## Discussion

Microarrays are a powerful method for detecting changes in gene expression and here we examine data from a range of different knockout mice. When comparing RNA from mutant mice and wild type controls, we observed a higher than expected frequency of changed genes near to the targeted/knocked-out gene. These genes were either clustered around the targeted allele or over-represented on that chromosome. Recent reports found evidence of linked changes in two knockouts [36], [37] (one of them published during the preparation of this manuscript). In our studies of PSD-95 and SAP102, the gene was disrupted in 129P2 ES cells and the RNA extracted from mice on a hybrid background (since a distinct mouse strain was used during breeding of the mutants). We consider the most likely explanation for the changes in gene expression of the genes clustered around the knockout locus to be that these genes were of ES cell origin (flanking DNA). In this model, the flanking DNA (from ES cells) contains polymorphisms that alter gene expression relative to the genetic background of the backcrossed mice. We found changes in gene expression in SAP102 mice (from 129P2 ES cells) backcrossed onto 129S5 and C57BL/6 strains. Although 129P2 and 129S5 might be closely related genetic backgrounds, a large variability has been reported between 129 substrains [38], [39]. The notion that flanking DNA can contribute to complications in mouse behavioural studies has been recognised and breeding strategies recommended to minimise its effects [40]. We have reported here that although backcrosses shorten the length of ES cell flanking regions more than ten backcrosses was insufficient to completely eliminate them (see PSD-95 and Foxa3 examples), as recognized in [40]. Marker selection (as the example provided by *hprt*) can accelerate the identification of mice with shorter flanking DNA.

An alternative model, which we cannot exclude, is that the changes in gene expression around the knockout locus were because of the modifications introduced during gene targeting (e.g. neomycin gene) that produce a *cis*-acting effect on neighbouring genes. This could arise either by disrupting a functional region in the host genome (i.e., a regulatory element) or interaction of the foreign sequence with the flanking DNA. The number of changing genes in the microarray experiments in the SAP102 and PSD-95 samples was inversely correlated with the number of backcrosses and active marker selection, suggesting the *cis*-acting effect, if it exists, only partially contributes. To explore a *cis* effect in more detail, a future approach could consist of analyzing microarray data from knock-in animals, where the insertion is made into a region lacking functional elements (i.e., coding region or a regulatory element).

Microarray analysis in knock-out animals should be carefully examined to avoid misinterpretation in the gene expression profile. Rather than assign the observed changes to the mutation, the genotype of the changed genes needs to be tested. This current study highlights the effects of flanking DNA of ES cell origin, which is selected during breeding of the mutant mice. It may therefore be necessary to genotype all changed genes before studying mutation-dependent effects. Although this may not be practical in all situations, the simplest approach would be to treat those changed genes on the targeted chromosome with extreme caution and only consider changes on other chromosomes or in those portions of the targeted chromosome estimated to segregate independently. Future array based methods could include mRNA profiling and a separate array for profiling the genotype of the corresponding probe sets and their surrounding regions. We would also recommend that when gene expression data is reported on mutant mice, that the chromosome location of the genes encoding those mRNAs is clearly reported. Studies on mutant mice on the inbred background of the ES cells would obviate the need to perform genotyping.

## Materials and Methods

### Animals

For microarray studies, the ages of mice used were as follows: 6 weeks old (129S5 strain); 12–13 weeks old (C57BL/6j strain). Number of mice: five SAP102^−/Y^ and five littermate wild-types (129S5 strain); four SAP102^−/Y^ and three littermate wild-types (C57BL/6j strain); five PSD-95^−/−^ and four littermate wild-types (hippocampus, C57BL/6j strain); two PSD-95^−/−^ and two littermate wild-types (cortex, C57BL/6j strain). Animals were dispatched by cervical dislocation. All dissections were performed consecutively on the same day at 4°C and tissue kept moist with chilled PBS (Gibco). Collected tissue (hippocampus and cortex) was immediately frozen in liquid nitrogen or immersed into RNAlater reagent (Qiagen) on ice for RNA isolation. Animals were treated in accordance with the UK Animal Scientific Procedures Act (1986).

### Nucleic acids isolation

RNeasy Mini and Midi columns (Qiagen) were used to purify the RNA from hippocampus and cortex, respectively. On-column DNase treatment was carried out to remove traces of DNA. During all the purifications, manufacturer's instructions were followed. Samples were concentrated with the addition of 1/10 vol of 3 M NaOAc pH 6 and 3 vol of 100% EtOH and precipitated at −20°C. Once resuspended in 12 µl of RNase-free H_2_O, an aliquot was used to check the RNA integrity in 1% formaldehyde-agarose gels.

To isolate genomic DNA, mouse tails were cut into 4–5 pieces and treated with 500 µl of the Lysis Buffer (10 mM Tris-HCl pH = 7.5, 1 mM EDTA, 1% SDS, 10 mM NaCl) and 2.5 µl Proteinase K (20 mg/ml) during 37 °C O/N. Then, 500 µl buffered phenol was added and the mix was incubated 1–2 h with gentle rotation at 4 °C. Once taking the supernatant after a low-speed spin (5,000 rpm 5 min), 500 µl of isoamylic-chloroform-phenol was used and spin at 13,000 rpm for 5 min. DNA was immediately precipitated by adding 1 ml isopropyl alcohol. After spinning at 13,000 rpm for 5 min, the pellet was washed with 500 µl 70% ethanol and resuspend in 100 µl H_2_O.

### Microarray hybridization, data acquisition and statistical analyses

For the transcriptome profiling, MG-430 2.0 arrays (Affymetrix) were used, that contain 45,101 probe sets including controls, one array per sample. Briefly, 3 µg of total RNA was reverse transcribed, labelled and hybridized using One-cycle Labelling Kit instructions (Affymetrix). Fluidics Station 450 and GCS3000, both from Affymetrix, were used for the washing and scanning steps respectively.

Signal intensities values and detection flags were extracted using GCOS (Affymetrix), using a Target value of 500 in the scaling step. Data were normalized by the median per gene and per array using GeneSpring v7 (Silicon Genetics): i) ANOVA test (p<0.05) without assuming equal variances, ii) signal intensity must be a Present or Marginal call in all wild-type samples when down-regulated and knock-out samples when up-regulated, and iii) fold change >1.8 that was selected arbitrarily for the purposes of the present work. In parallel data was processed using dChip (PM/MM difference model) [41]. Only those probe sets appearing in both analyses were considered.

The significant genes obtained in the microarray analyses were grouped in bins of 10 Mb to simplify both visualization in the figures and χ^2^ tests. Centromeres were not considered. We compared the distribution of the significant genes along the targeted chromosome to a random distribution. This random distribution was calculated by dividing the number of significant genes by the number of bins. When testing the distribution of significant genes between all the chromosomes, three types of random distribution were analyzed: equal proportion of significant genes in all the chromosomes, proportion normalized by the length of each chromosome and proportion normalized by the number of genes (based upon Ensembl release 45). The microarray data is deposited in the ArrayExpress database, www.ebi.ac.uk/arrayexpress, accession number E-MEXP-1159.

### Retrotranscription and PCR

Typically, 1 µg of total RNA was used for the retrotranscription, following the recommendations of Invitrogen (SuperScript II Reverse Transcriptase). PCR reactions were set up per sample using 1 µl cDNA, 0.3 µM each primer, 25 µl 2× Quantitect SYBR Green PCR Master Mix (Qiagen) to a final volume of 50 µl. The sequences of the primers were 5′-GTGGATTCTTTCTCCTTCAGC-3′ and 5′-ATACAACCTCCTCAGCTTTGC-3′ (Fundc2), 5′-TTCTGGTTTGTTTTGTTTTGC-3′ and 5′-TTCATGCCTTTTGTAAGCAAG-3′ (BB315069), 5′-CTCTCCACTTCATGCTCCAC-3′ and 5′-ATGGACATCCAGATTGCTAAG-3′ (Fbxo39), 5′-AATCGTCATAGGGAGGTTCAC-3′ and 5′-AGACCTTGCTATAGGCTCCAG-3′ (BG092359), 5′-ACGAGAGTGGTCAAGGTTAAAG-3′ and 5′-GGAGAGAAGATCATCGTTGG-3′ (PSD-95), 5′-AAAGGACCTCTCGAAGTGTTG-3′ and 5′-ATTTGGCTTTTCCAGTTTCAC-3′ (Hprt), 5′-GTGCTACGGGTGAATGAG-3′ and 5′-TGGTCTATCTGAAGGTGGCC-3′ (Sap102). Reactions were run out in the 7500 Real Time PCR System (Applied Biosystems) with the following conditions: 1 cycle of 95°C for 15 min, 35–40 cycles of 94°C for 30 s, 55°C for 30 s, 72°C for 50 s, 1 cycle of 72°C for 5 min. The resulting amplicons were checked for specificity and size in agarose gels. The data generated was analysed in 7500 System SDS v1.2.2 software, that calculated the cycle threshold (C_T_) and the fold change ( = 2^−ΔΔCT^). GAPDH was used as endogenous controls. To analyze statistically the quantitative PCR results, Student's t-test was used.

For genotyping, each PCR was performed using 1 µl genomic DNA, 1 U Platinum Taq DNA polymerase (Invitrogen), 5 µl PCR buffer 10× (Invitrogen), 10 mM each dNTP, 50 mM MgCl_2_ and 0.5 µM each primer in a final volume of 50 µl. For *hprt* alleles genotyping, the protocol described in [30] was used with few modifications. Reactions to detect wild-type and mutant alleles were set up in parallel for the same genomic DNA: 1 cycle of 94°C 5 min, 35 cycles of 94°C 30 s, 62°C 30 s for the wild-type allele or 67°C for the null allele, 72°C 60 s and 1 cycle of 72°C 5 min. Addition of 1% DMSO was required for the null allele genotyping. For *atp7a* allele genotyping, the protocol described in [31] was used with few modifications, in which the conditions were 1 cycle of 94°C 5 min, 35 cycles of 94°C 30 s, 55°C 30 s, 72°C 60 s and 1 cycle of 72°C 5 min.
